# Exploring cognitive, behavioral and autistic trait network topology in very preterm and term-born children

**DOI:** 10.3389/fpsyg.2023.1119196

**Published:** 2023-04-27

**Authors:** Marguerite Leoni, Lucy D. Vanes, Laila Hadaya, Dana Kanel, Paola Dazzan, Emily Simonoff, Serena J. Counsell, Francesca Happé, A. David Edwards, Chiara Nosarti

**Affiliations:** ^1^Centre for the Developing Brain, School of Biomedical Engineering & Imaging Sciences, King’s College London, London, United Kingdom; ^2^Department of Child and Adolescent Psychiatry, Institute of Psychiatry Psychology and Neuroscience, King’s College London, London, United Kingdom; ^3^Department of Psychological Medicine, Institute of Psychiatry, Psychology and Neuroscience, King's College London, London, United Kingdom; ^4^South London and Maudsley NHS Foundation Trust and King's College London, National Institute for Health Research (NIHR) Mental Health Biomedical Research Centre, London, United Kingdom; ^5^Social, Genetic and Developmental Psychiatry Centre, Institute of Psychiatry Psychology and Neuroscience, King’s College London, London, United Kingdom

**Keywords:** very preterm birth, network analysis, temperament, executive function, emotion regulation, autism

## Abstract

**Introduction:**

Compared to full-term (FT) born peers, children who were born very preterm (VPT; <32 weeks’ gestation) are likely to display more cognitive and behavioral difficulties, including inattention, anxiety and socio-communication problems. In the published literature, such difficulties tend to be studied independently, thus failing to account for how different aspects of child development interact. The current study aimed to investigate children’s cognitive and behavioral outcomes as interconnected, dynamically related facets of development that influence one another.

**Methods:**

Participants were 93 VPT and 55 FT children (median age 8.79 years). IQ was evaluated with the Wechsler Intelligence Scale for Children—4^th^ edition (WISC-IV), autism spectrum condition (ASC) traits with the social responsiveness scale—2^nd^ edition (SRS-2), behavioral and emotional problems with the strengths and difficulties questionnaire (SDQ), temperament with the temperament in middle childhood questionnaire (TMCQ) and executive function with the behavior rating inventory of executive functioning (BRIEF-2). Outcome measures were studied in VPT and FT children using Network Analysis, a method that graphically represents partial correlations between variables and yields information on each variable’s propensity to form a *bridge* between other variables.

**Results:**

VPT and FT children exhibited marked topological differences. *Bridges* (i.e., the variables most connected to others) in the VPT group network were: conduct problems and difficulties with organizing and ordering their environment. In the FT group network, the most important *bridges* were: difficulties with initiating a task or activity and prosocial behaviors, and greater emotional problems, such as lower mood.

**Discussion:**

These findings highlight the importance of targeting different aspects of development to support VPT and FT children in person-based interventions.

## 1. Introduction

Compared to their full-term born peers (FT; 38–42 weeks’ gestation), children who were born very preterm (VPT; <32 weeks’ gestation) display greater behavioral difficulties, such as inattention, emotional dysregulation, socio-communication problems, anxiety, and internalizing behaviors (Rothbart et al., 2007; Johnson and Marlow, 2011; Arpi and Ferrari, 2013; Burnett et al., 2013; Brydges et al., 2018). VPT children also exhibit increased autism spectrum condition (ASC)-like traits (Treyvaud et al., 2013) and are more likely to receive an autism diagnosis compared to their term-born peers (7% vs. 1.5%, respectively) (Agrawal et al., 2018).

Studies focusing on ASC traits in VPT and extremely preterm (EPT; <28 weeks’ gestation) samples have revealed a distinct phenotypic expression from that observed in FT children, suggesting a preterm-specific ASC traits aetiology (Verhaeghe et al., 2016; Bröring et al., 2018; Chen et al., 2019). ASC trait aetiology in VPT/EPT children appear to be primarily rooted in poor socio-emotional abilities and difficulties with social communication and interaction (Johnson and Marlow, 2011; Jaekel et al., 2013; Johnson et al., 2018), rather than a combination of social communication and interactions problems, and rigid and repetitive behaviors and interests, as observed in FT samples. Moreover, social communication and interactions impairments appear to be more homogenously distributed across VPT compared to FT populations, who display greater behavioral heterogeneity (Chen et al., 2019), thus supporting the idea of a preterm-specific ASC trait aetiology.

VPT children are not only more likely to display elevated ASC traits compared to FT-born controls, but also exhibit broader developmental difficulties. A within-group stratification study conducted by Johnson et al. (2018) explored distinct patterns of cognitive and behavioral development in 2 year-old preterm children, and identified a “non-optimal” subgroup characterized by ASC symptomatology, poorer cognitive and language skills, greater social, emotional and attention difficulties, and heightened risk of developing behavioral problems. Indeed, VPT-born children differ on measures of temperament, displaying shorter attention span, poorer ability to focus, and higher rates of activity compared to their FT-born peers (Cassiano et al., 2020), which underlie externalizing behavior (Bora et al., 2014). VPT-birth has also been associated with greater cognitive difficulties, with VPT children showing lower intellectual quotient, working memory, verbal abilities and processing speed scores than their FT-born peers (Allotey et al., 2017). However, it is unclear how these difficulties identified in VPT children interact or influence one another, highlighting the need to investigate multiple cognitive and behavioral outcomes as a set of interconnected, dynamically interacting facets of development. Indeed, such a paradigm could allow for the identification of core cognitive-behavioral features to better understand preterm-specific ASC symptomatology (Borsboom and Cramer, 2013; Eadeh et al., 2021), in alignment with a (multi-)dimensional and trait-based approach to ASC (Constantino, 2009; Volkmar and McPartland, 2015; Constantino and Charman, 2016). Exploring a broad range of developmental outcomes impacted by very preterm birth in a single model, may allow us to delineate preterm-specific developmental correlates of ASC traits.

Previous studies have applied network analysis to ASC populations, studying the network topology of ASC and other behaviors that tend to be comorbid with ASC (Anderson et al., 2015; Ruzzano et al., 2015; Montazeri et al., 2019). These studies have increased our understanding of the dynamic interactions between ASC features and of pathways to outcome phenotypes. Network analysis is a statistical approach which uses nodes (i.e., variables indexing psychological/behavioral constructs) and edges (i.e., partial correlations between nodes) to map the interaction between psychological constructs, and evaluate how variables correlate with one another. Contrary to a correlation matrix, network analyses allow for a graphical representation of the degree and pathways of interaction, allowing for the most important variables underlying specific phenotypes to be identified.

To our knowledge, no prior study has used network analysis to explore multiple facets of development following VPT birth in mid-childhood. This developmental stage is particularly important, as evidence suggests that during early life it is complex to differentiate between ASC and ADHD traits and behavioral and temperamental difficulties (Shephard et al., 2019), hence a network analysis approach may be particularly useful in delineating complex phenotypes. In the present study we use network analysis to investigate ASC traits in the context of other behavioral and cognitive measures, in order to understand whether structural relationships between these outcome phenotypes differ between VPT and FT children. One of the advantages of this approach is that it allows us to explore how different developmental domains, previously analysed independently in VPT children, interact with one another in a single model. We focus on *bridge symptoms*, or nodes connecting different disorders or constructs (Jones et al., 2021), to understand comorbidity and shared characteristics between psychopathology, behavioral, emotional or temperamental difficulties.

The current study places a particular focus on ASC traits when comparing FT and VPT networks. While this study is mostly exploratory, we hypothesized that, in addition to VPT participants displaying greater ASC traits, behavioral, cognitive and temperamental difficulties compared to their FT peers, they would show a topologically different cognitive-behavioral network architecture. Specifically, we hypothesized that, similar to findings pertaining to VPT toddlers (Johnson et al., 2018), ASC traits would be more strongly associated with emotional and temperamental difficulties in VPT compared to FT children (Vlaeminck et al., 2020).

## 2. Methods

### 2.1. Participants

Very preterm participants, born before 32 completed weeks of gestation, were recruited as new-borns between 2010 and 2013 as part of the Evaluation of Preterm Imaging study (ePrime; EudraCT 2009-011602-42; Edwards et al., 2018), from hospitals within the North and Southwest London Perinatal Networks and were followed up behaviorally throughout childhood (Kanel et al., 2021a; Hadaya et al., 2022). The current study focuses on behavioral assessments conducted at a median age of 8.75 years (*n* = 148). Control participants, born after 37 completed weeks of gestation, were recruited from the local community (median age = 8.83 years; *n* = 55). Participants’ socio-demographic and clinical characteristics are presented in Table 1. There was no significant difference in gestational age [*t*(153) = −0.77, *p* = 0.44, confidence interval (CI) = (29.27;29.49)] or sex [*t*(307) = −0.63, *p* = 0.53, CI = (−0.13;0.07)] between the VPT participants assessed at 4–7 years (Kanel et al., 2021b) and those included in the current study. There was however a significant difference IMD rank [*t*(178) = −3.99, *p* < 0.001, CI = (−0.92;-0.31)], with VPT participants in the current sample being significantly more deprived than those assessed at 4–7 years. Control participants were only included as part of the current study, hence the data at previous time points are unavailable. Exclusion criteria were major congenital malformation, contraindication to magnetic resonance imaging (data not shown here), caregiver(s) unable to speak English or the participant being under child protection proceedings. Ethical approval for the study was granted by London South East Research Ethics Committee (REC: 19/LO/1940) and London Stanmore Ethics Committee (REC: 18/LO/0048).

**Table 1:** Socio-demographic and clinical charactersitics of the study sample.

		VPT (*N* = 93)	FT controls (*N* = 55)
Gestational age at birth (weeks)	*Median*	29.86	40
	*IQR*	[27.57–31.86]	[39–40.86]
Age at testing	*Median*	8.75	8.83
	*IQR*	[8.33–9.17]	[8.25–9.17]
Sex	*Male*: *Female*	50: 43	23: 32
	*%*	53.7, 46.3%	41.8, 58.2%
Ethnicity (N)	White	43	40
	Mixed/multiracial	7	4
	Asian/Asian British	16	2
	Black/African/Caribbean/Black British	9	1
	Other ethnic origin	5	0
IMD score quintile (N)	1 (least deprived)	5	2
	2	18	17
	3	22	7
	4	21	9
	5 (most deprived)	27	20

Ethnicity: The groups were characterized according to the Office of National Statistics (Office for National Statistics, 2016; https://www.ons.gov.uk/ethnicity/): White (English/Welsh/Scottish/Northern Irish/British, Irish, Any other White background); Mixed/Multiple ethnic groups (White and Black Caribbean, White and Black African, White and Asian, Any other Mixed/Multiple ethnic background); Asian/Asian British (Indian, Pakistani, Bangladeshi, Chinese, Any other Asian background); Black/African/Caribbean/Black British (African, Caribbean, Any other Black/African/Caribbean background); Other ethnic group (Arab, Any other ethnic group).

IMD, index of multiple deprivation, a measure of participant’s socio-economic status (Department for Communities and Local Government, 2011; https://tools.npeu.ox.ac.uk/imd/).

### 2.2. Assessment of outcome measures

#### 2.2.1. Cognitive assessment

Participants were administered the Wechsler Intelligence Scale for Children Fourth Edition (WISC-IV; Wechsler, 2003), to assess general intellectual development. The WISC-IV provides a full-scale IQ (FSIQ) score and comprises four subscales evaluating narrower cognitive functioning: *verbal comprehension*, *perceptual reasoning*, *processing speed* and *working memory*. Raw scores were transformed into age-normed scaled scores.

#### 2.2.2. Parent-report questionnaires

Participants’ caregiver(s) were asked to complete the following questionnaires.

The Social Responsiveness Scale Second edition (SRS-2, Constantino and Gruber, 2012) assessed ASC symptomatology with 65 items comprising two subscales: *rigid and repetitive behaviors* (RRB), and *social communication and interaction* (SCI), aligned with the two main symptom domains of the latest diagnostic manual for ASC (DSM-V; American Psychiatric Association, 2013). Each item is scored along a 4-point Likert scale and raw scores are transformed into *T*-scores. A score of 76 or higher on the SRS-2 is associated with a clinical diagnosis of ASC (Constantino and Gruber, 2012).

The strengths and difficulties questionnaire (SDQ, Goodman, 2001), assessed child behavior using five subscales indexing conduct, peer relation problems, emotional problems, prosocial behaviors and a measure of ADHD focusing on hyperactivity-inattention. Responses are given on a three-point Likert scale ranging from 0: “not true” to 3: “certainly true.”

The Temperament in Middle Childhood Questionnaire (TMCQ) for children aged seven to 10 (Simonds and Rothbart, 2004) was administered to measure child temperament. It comprises 157 items and responses range from 1: “almost always untrue” to 5: “almost always true,” with an additional “does not apply” option. These items yield three subscales: *negative affectivity* (NA), *effortful control* (EC) and *surgency* (SU).

Finally, the Behavior Rating Inventory of Executive Function Second Edition (BRIEF-2; Gioia et al., 2015), a 63-item questionnaire, measured participants’ executive functioning in everyday settings. The BRIEF-2 has nine sub-scale scores: *inhibition*, *self-monitoring*, *shift*, *emotional contro*l, *initiating behaviors*, *working memory*, *planning and organizing*, *task monitoring* and *organization of materials*.

### 2.3. Data analysis

Statistical analyses were conducted using R and RStudio (version 1.3.1).

#### 2.3.1. Univariate group comparisons

Independent samples t-tests were computed to test for differences between VPT participants and FT controls on each outcome measure. All *p* values reported are corrected for multiple comparisons controlling for false discovery rate (FDR).

In addition, we tested whether VPT participants had greater ASC-like difficulties (*RRB* or *SCI*) compared to their FT born peers, after controlling for sex, age at assessment, and IQ using separate multiple regression models.

#### 2.3.2. Network analysis

Network analyses were conducted using the Gaussian Graphical Model (Lauritzen, 1996) with the *glasso* function, included in the qgraph R package (Epskamp et al., 2012). Separate networks were estimated for the FT and VPT groups, respectively. All subscale outcome measures of interest (verbal comprehension, perceptual reasoning, working memory and processing speed scores from the WISC-IV; inhibition, self-monitoring, shift, emotional control, initiating behavior, working memory, planning and organizing, task monitoring, organization of material scores from the BRIEF-2; social awareness, social cognition, social communication, social motivation, rigid and repetitive behaviors as measured by the SRS-2; surgency, effortful control and negative affect scores from the TMCQ; and emotional problems, conduct problems, hyperactivity, peer relationship problems, and prosocial behavior scores measured by the SDQ) were included and equally weighted.

For both the FT and VPT group networks, the following measures were computed for each node: *bridge closeness*, referring to the average distance between one node and any other node that is part of another construct (i.e., questionnaire or assessment); *bridge betweenness*, referring to the number of times a node lies on the shortest path between two other nodes that are from different constructs; *bridge expected influence (EI)*, referring to the sum of all the edges, either negative or positive, between each node and all other nodes from different constructs (Cramer et al., 2010; Epskamp et al., 2012; Eadeh et al., 2021; Jones et al., 2021).

To test group invariance of the two networks regarding their overall structure, *global strength* (i.e., the sum of all edge weights) and specific *edges*, a network comparison test was computed using the *NetworkComparisonTest* R package (Dalege et al., 2017), which involves a bootstrapping procedure that facilitates the comparison of networks with unequal sample sizes. Given our focus on ASC-like traits, we conducted comparisons between the SRS-2 subscale measures and all other nodes. Edge comparisons across both networks, between SRS-2 subscale measures and all other measures were Bonferroni corrected.

## 3. Results

### 3.1. Sample characteristics and univariate group comparisons

Socio-demographic and clinical characteristics of the sample are given in Table 1. There was no significant difference in sex [*χ*^2^(1,141) = 1.52, *p* = 0.217] or age [*t*(92) = 0.23, 95% confidence interval = (−0.2;0.3); *p* = 0.822] between the VPT and FT groups.

Mean differences in outcome scores between VPT participants and FT controls are shown in Table 2. In summary, VPT participants had significantly lower IQ and TMCQ effortful control scores, but displayed higher ASC traits, SDQ emotional and behavioral difficulties, and TMCQ negative affectivity scores, and had significantly greater executive function difficulties compared to controls (i.e., BRIEF-2 scores). The mean group difference with the greatest effect size was on ASC-like traits, measured by the SRS-2. Importantly, after controlling for age at testing, sex, full scale IQ, and IMD rank, group differences remained significant for both SRS-RRB [*beta* = 4.46, *t*(118) = 2.57, 95% CI = (1.0;7.9); *p* = 0.011] and SRS-SCI [*beta* = 5.26, *t*(118) = 2.86, 95% CI = (1.6;8.9), *p* = 0.004]. Furthermore, 12 of the 93 VPT participants (12.9%) and 2 of the 55 controls (3.64%) had an SRS-2 score equal to or greater than 76 [*χ*^2^(41,141) = 60.7, *p* = 0.024].

**Table 2:** Descriptive statistics for all outcome measure in the VPT and FT control groups.

	VPT (*N* = 93)		FT controls (*N* = 55)		*T*	Cohen’s *d*	95%CI
	Mean (SD)	Range	Mean (SD)	Range			
WISC-IV FSIQ	103.74(14.59)	(68–137)	111.2(12.89)	(81–141)	*t*(125) = 3.22**	0.53	[2.9; 12.1]
VC	105.36(13.3)	(75–132)	108.31(13.8)	(83–140)	*t*(110) = 1.27	0.22	[−1.66; 7.55]
PR	103(17.4)	(10–135)	111.8(15.84)	(67–143)	*t*(122) = 2.95*	0.49	[2.72; 13.84]
PS	99.42(14.86)	(68–136)	107.96(14.41)	(70–131)	*t*(120) = 3.43***	0.58	[3.61; 13.48]
WM	98.65(15.81)	(50–141)	104.96(12.3)	(71–135)	*t*(135) = 2.7*	0.43	[1.69; 10.93]
SRS-2	59.47(14.04)	(39–90)	49.44(8.62)	(38–79)	*t*(138) = −5.25***	−0.82	[−13.8; −6.3]
SCI	54.82(11.39)	(39–90)	47.61(8.08)	(36–81)	*t*(136) = −4.38***	−0.7	[−10.45;-3.95]
RRB	53.85(11.41)	(41–90)	47.26(6.57)	(38–78)	*t*(138) = −4.35***	−0.67	[−9.59; −3.59]
SDQ	11.47(5.34)	(1–26)	9.05(4.44)	(0–25)	*t*(130) = −2.93**	−0.48	[−4.0; −0.8]
Emo	2.33(2.24)	(0–9)	1.67(1.77)	(0–9)	*t*(133) = −1.96	−0.32	[−1.33; 0.01]
Cond	1.73(1.84)	(0–9)	1.14(1.37)	(0–5)	*t*(137) = −2.18*	−0.35	[−1.11; −0.05]
Peer Rel	3.16(1.66)	(0–8)	2.53(1.33)	(0–6)	*t*(132) = −2.55*	−0.42	[−1.14; −0.14]
HyperA	4.23(1.93)	(0–8)	3.7(1.94)	(0–8)	*t*(114) = −1.49	−0.27	[−1.18; 0.13]
ProSoc	8.42(1.92)	(1–10)	8.8(1.7)	(0–10)	*t*(125) = 1.21	0.2	[−0.24; 0.98]
BRIEF-2	105.16(25.0)	(60–178)	92.58(20.16)	(66–158)	*t*(128) = −3.26**	−0.54	[−20.21; −4.94]
Beh	20.46(5.73)	(12–35)	17.19(4.97)	(12–34)	*t*(121) = −3.56**	−0.6	[−5.09; −1.45]
Emo	25.94(7.97)	(16–48)	23.15(6.22)	(16–43)	*t*(129) = −2.31*	−0.38	[−5.18; −0.4]
Cog	58.76(14.45)	(32–95)	52.24(12.1)	(35–81)	*t*(125) = −2.87*	−0.48	[−11; −2]
TMCQ	2.95(0.23)	(2.3–3.7)	2.92(0.18)	(2.4–3.2)	*t*(135) = −0.83	−0.13	[−0.08; 0.04]
NegA	2.4(0.65)	(1.1–4.1)	2.17(0.55)	(1.2–3.4)	*t*(128) = −2.34*	−0.39	[−0.44; −0.04]
EffC	3.28(0.45)	(2.2–4.4)	3.52(0.4)	(2.6–4.7)	*t*(127) = 3.45**	0.57	[0.11;0.4]
Surg	3.17(0.47)	(1.9–4.1)	3.08(0.45)	(1.8–4.1)	*t*(119) = −1.23	−0.21	[−0.25;0.06]

WISC, Wechsler intelligence scale for children; FSIQ, full scale IQ; VC, verbal comprehension; PR, perceptual reasoning; PS, processing speed; WM, working memory; SRS-2, social responsiveness scale; SCI, social communication and interaction; RRB, rigid and repetitive behaviors; SDQ, strengths and difficulties questionnaire; Emo, emotional difficulties; Cond, conduct problems; Peer Rel, peer relationship difficulties; HyperA, hyperactivity; ProSoc, prosocial behaviors; Int, initiating behaviors; Ext, externalizing behaviors; BRIEF-2, behavior rating inventory of executive functioning, second edition; Beh, behavioral difficulties; Emo, emotional difficulties: Cog, cognitive difficulties; TMCQ, temperament in middle children questionnaire; NA, negative affectivity; EC, effortful control; SU, surgency.

* = *p* < 0.05; ** = *p* < 0.01; *** = *p* < 0.001.

### 3.2. Network analysis

#### 3.2.1. Network estimation

**Figure 1:**
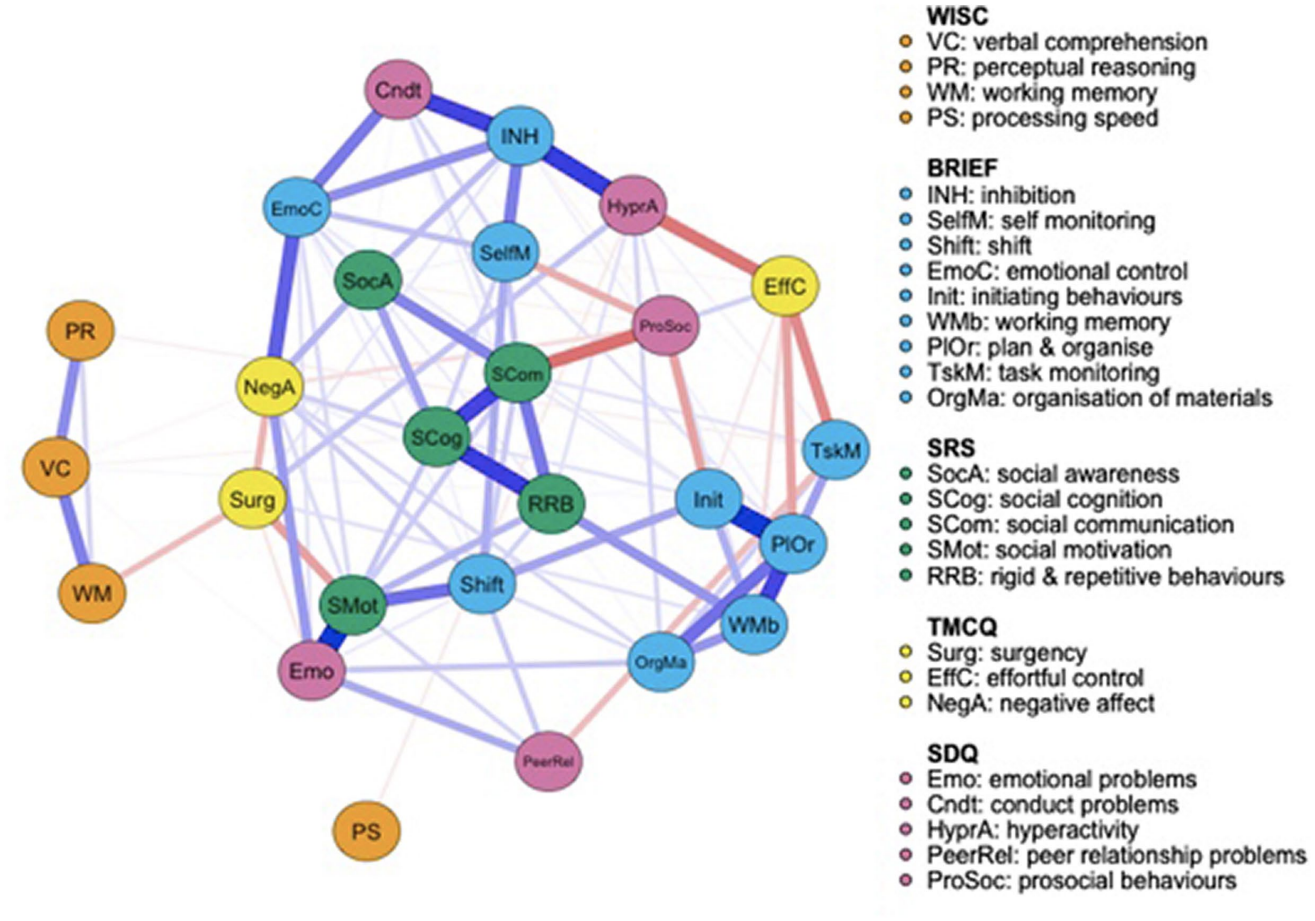
Full term control group network structure of autism spectrum condition (ASC) symptomatology and cognitive, executive functioning, temperament and psychopathology outcome variables. Networks consist of round elements (nodes) which correspond to variables. Lines connecting each node (edges) represent partial correlations between nodes.

**Figure 2:**
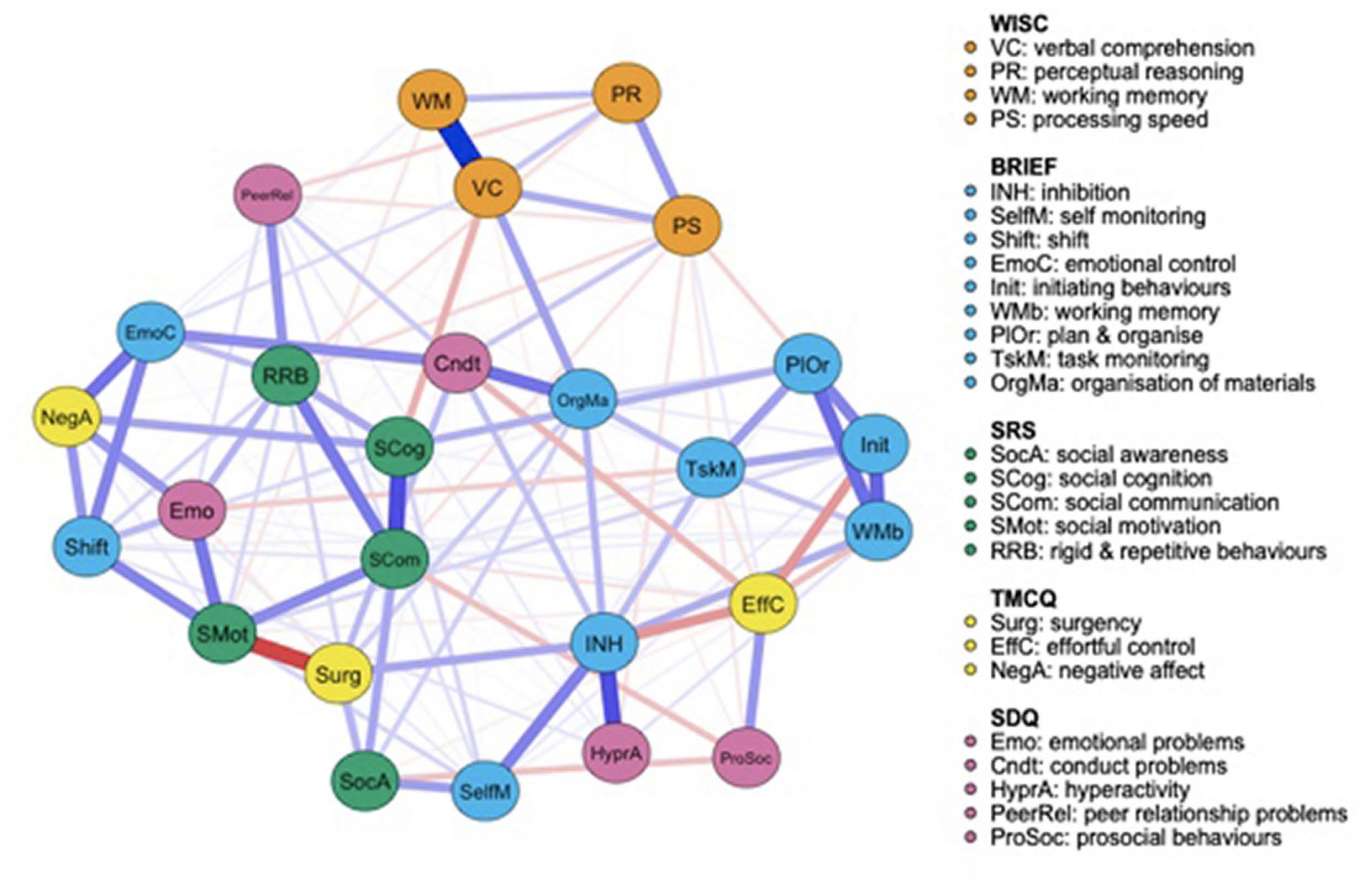
Very preterm group network structure of autism spectrum condition (ASC) symptomatology and cognitive, executive functioning, temperament and psychopathology outcome variables.

Both the FT (Figure 1) and the VPT control group (Figure 2) networks had 26 nodes. Edges reveal the strength of a relationship between two nodes, or variables, which can be either positive (represented by a blue line), or negative (represented by a red line). The strength of this association is graphically depicted by the thickness of the line, with thicker edges representing statistically stronger associations. Of the 325 possible edges, the VPT network showed 136 (41.84%) that were non-zero, with an overall edge mean weight of 0.02. The FT control group network had 109 non-zero edges (33.54%), with a similar mean weight of 0.02.

#### 3.2.2. Qualitative relationship between variables

Figure 1 displays the FT controls’ network. Visually, while the WISC-IV measures appear less connected to the rest of the network, the SRS-2 scores cluster together and the BRIEF-2 is split into two broader categories: one relating to emotional and behavioral regulation, and the other focused more specifically on cognitive control. Social motivation scores are strongly positively associated with emotional problems and moderately negatively associated with surgency, suggesting that greater difficulties with social motivation are associated with greater emotional problems and lower levels of surgency. SRS-2 subscale scores clustered together. Social communication is strongly negatively associated with prosocial behaviors and strongly positively associated with social cognition, but only weakly positively correlated with social awareness. Prosocial behaviors measured by the SDQ are also negatively associated with initiating behaviors and self-monitoring, both measured by the BRIEF-2.

Figure 2 presents the VPT participants’ network. Relative to the FT group network, all measures seem to be closely connected connected to one-another. Social cognition, as measured by the SRS-2, shows a moderate negative association with verbal comprehension, measured by the WISC-IV, and a strong positive correlation with social communication, also measured by the SRS-2, suggesting that social cognition difficulties are associated with poorer verbal comprehension abilities and greater social communication difficulties. SRS-2 social motivation is strongly negatively associated with surgency, measured by the TMCQ, but positively associated with emotional problems (SDQ), social communication (SRS-2) and attention shift (BRIEF-2). Prosocial behaviors are weak-to-moderately negatively associated with social awareness. SRS-2 subscale scores appear less clustered together in the VPT compared to the FT control group network. To ease interpretability of results, correlation matrices for all outcome variables included in the very preterm (VPT) and FT control network are also provided in the Supplementary material, Figures S1 and S2, respectively.

#### 3.2.3. Node and bridge centrality

**Figure 3:**
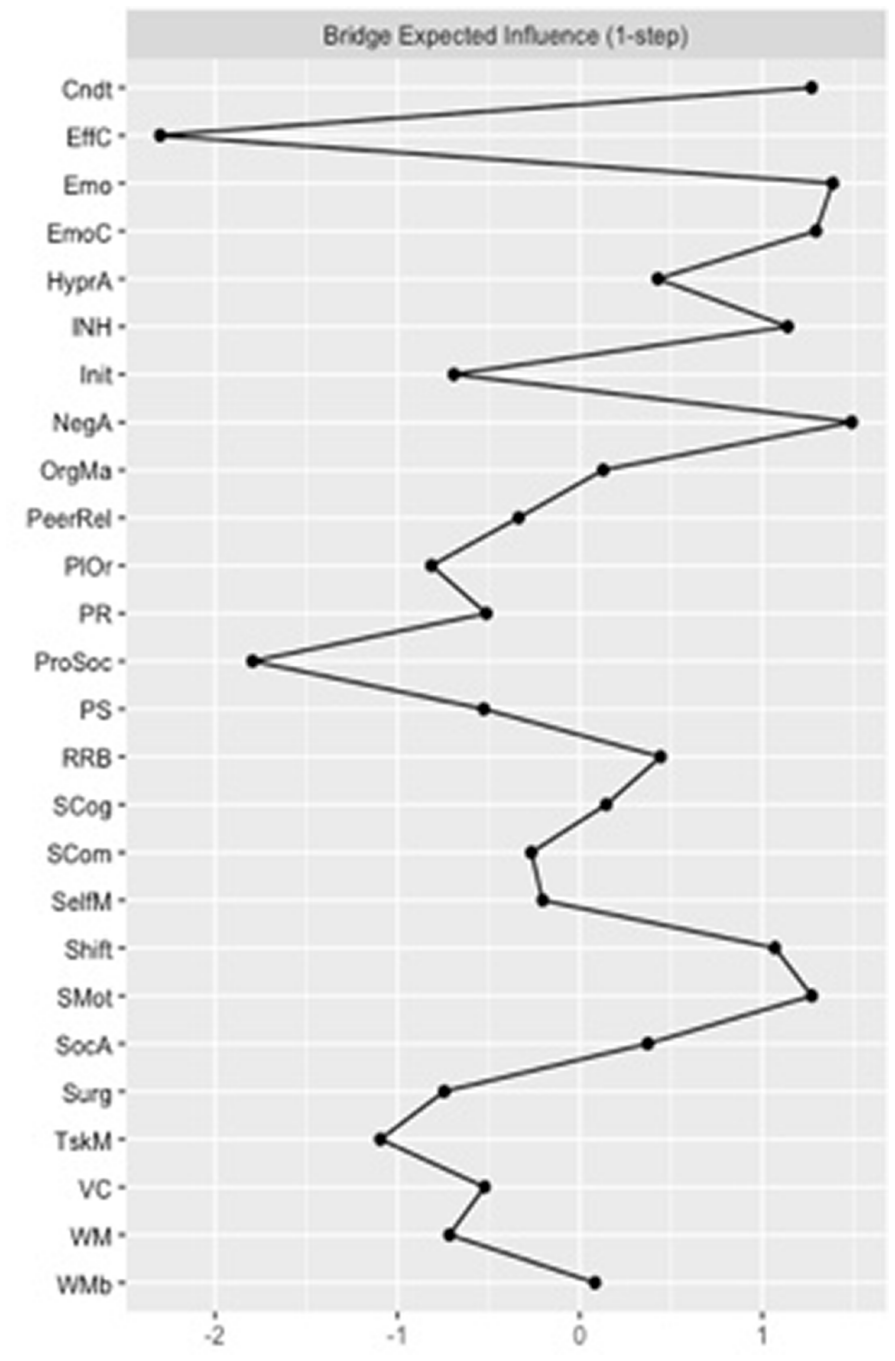
Bridge expected influence for the full-term (FT) control group network. Cndt, conduct problems; EffC, effortful control; Emo, emotional problems; EmoC, emotional control; HyperA, hyperactivity; INH, inhibition; Init, initiating behaviors; NegA, negative affectivity; OrgMa, organizing materials; PeerRel, peer relationship problems; PlOr, planning and organizing; PR, perceptual reasoning; ProSoc, prosocial behaviors; PS, processing speed; RRB, rigid and repetitive behaviors; SCog, social cognition; SCom, social communication; SelfM, self-monitoring; SocA, social awareness; Surg, surgency; TskM, task monitoring; VC, verbal comprehension; WM, working memory; WMb, working memory measured by the BRIEF-2.

Quantitatively, focusing on the bridge centrality of the FT control network, “effortful control” and “prosocial behaviors” had the highest *bridge closeness* scores, suggesting that these two measures showed the shortest average distance to all other nodes outside their domains. “Initiating behaviors” and “planning and organizing” had the highest *bridge betweenness* values, showing that measures more frequently lie on the shortest path between two other measures, pointing towards a potential role in serving as connection points (or “middlemen”) between the other two constructs. “Negative affect” and “emotional problems” were the nodes with the highest *bridge expected influence* (Figure 3), suggesting that these nodes were the most important in connecting different behavioral, temperamental, cognitive and ASC-trait constructs.

**Figure 4:**
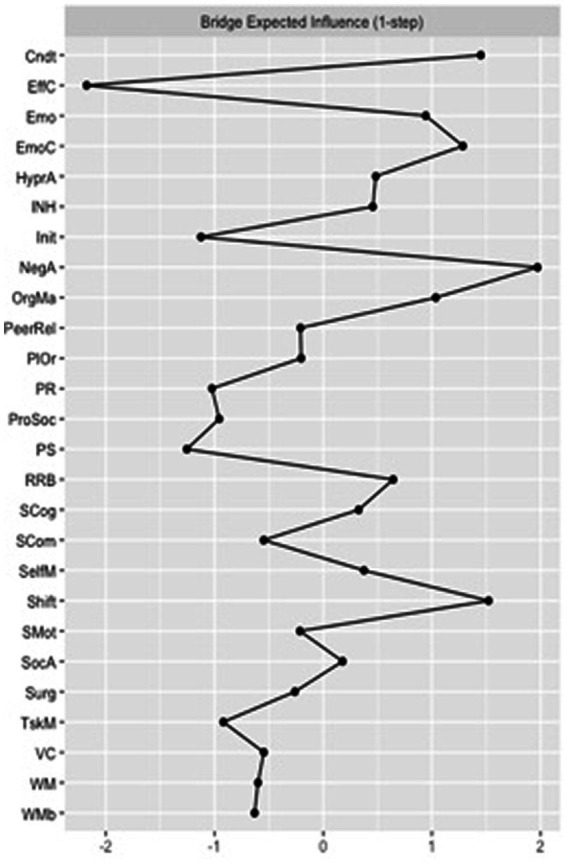
Bridge expected influence for the very preterm (VPT) group network. Cndt, conduct problems; EffC, effortful control; Emo, emotional problems; EmoC, emotional control; HyperA, hyperactivity; INH, inhibition; Init, initiating behaviors; NegA, negative affectivity; OrgMa, organizing materials; PeerRel, peer relationship problems; PlOr, planning and organizing; PR, perceptual reasoning; ProSoc, prosocial behaviors; PS, processing speed; RRB, rigid and repetitive behaviors; SCog, social cognition; SCom, social communication: SelfM, self-monitoring; SocA, social awareness: Surg, surgency; TskM, task monitoring; VC, verbal comprehension; WM, working memory; WMb, working memory measured by the BRIEF-2.

Regarding the bridge centrality estimates for the VPT network, “organization of materials” and “conduct problems” were the nodes with the greatest *bridge closeness.* These two nodes were also the ones with the greatest *bridge betweenness* scores. Finally, “negative affect” and “shift” were the nodes with the greatest *bridge expected influence* scores (Figure 4).

#### 3.2.4. Network comparison

There was a significant difference in global strength, *p* = 0.036 and edge weight, *p* = 0.020 between the overall structure of the VPT and the FT network, with nodes in the VPT group network being more tightly associated with one-another, compared to those in the FT group.

We focused on SRS-2 subscale scores, in order to explicitly compare how these relate to other characteristics in both groups. Table 3 presents edge value comparisons that show significant differences between the VTP and FT networks. SRS-2 subscale scores were more connected to other variables in the VPT compared to the FT network. Specifically, VPT participants showed a stronger positive association between “social cognition” and “plan and organize” compared to FT participants, with a poorer ability to interpret others’ emotions and intentions, being associated with a poorer ability to plan and organize tasks or the environment. The VPT group also showed a stronger negative association between “social motivation” and “working memory,” as measured by the WISC-IV, indicating that poorer motivation to engage in social interactions is associated with greater working memory difficulties. The positive connection between “social communication” and “social motivation” was significantly stronger in the VPT compared to the FT network. Finally, the negative correlation between “social motivation” and “surgency” was also significantly stronger in the VPT compared to the FT network, indicating that reduced levels of motivation to engage in social situations is associated with lower levels of sociability and positive affect. Despite applying a Bonferroni correction to the edge comparisons, the unequal sample size across both groups constitutes a limitation to our network comparison.

**Table 3:** Comparison between the very preterm (VPT) and full-term (FT) networks in edge values between SRS-2 subscale scores and all other nodes.

Edges	VPT network Edge value	FT network Edge value	*p*, edge comparison
Social cognition—plan and organize	0.14	0	0.017*
Social motivation—working memory	−0.04	0	0.002**
Social communication—social motivation	0.21	0.08	0.036*
Social motivation—surgency	−0.34	−0.14	0.012*

* = *p* < 0.05; ** = *p* < 0.01; *** = *p* < 0.001.

Only significant results are displayed.

## 4. Discussion

In this study we used network analysis to explore how ASC traits, cognitive, behavioral and temperament outcomes dynamically interact in VPT and FT born children. VPT participants displayed elevated ASC traits compared to their FT peers, with group differences in the SRS-2 subscales showing the largest effect sizes of all behavioral measures. Subsequent network analyses allowed us to further investigate the underlying mechanisms that may be driving these behavioral differences. The exploratory nature of this approach allowed us to uncover a number of novel associations that have not previously been observed in the published literature. Compared to the FT group, VPT participants’ ASC traits, cognitive, behavioral and temperament outcomes seemed to be predominantly centered around and driven by conduct and executive function difficulties, in particular shifting attention and organizing materials. In comparison, the FT participants’ behavioral, cognitive and temperamental profiles appeared to be associated with difficulties in initiating behaviors, emotional regulation, and to a lesser degree, executive function abilities compared to the VPT children.

While the main focus of this study was to explore how ASC traits and cognitive, behavioral and temperament outcome measures relate to one-another in VPT and FT groups, the findings regarding increased conduct problems in VPT compared to FT children are unexpected. This is because previous research has suggested that conduct problems may not be elevated in VPT compared to FT children (Wolke, 1998; Johnson et al., 2010). A direct network comparison indicated that in VPT children, conduct problems were more closely linked to constructs from other domains other than ASC traits, acting as a bridge between constructs, such as emotional control and organizing their environment. These findings suggest that where conduct problems do arise in VPT children, these may be more tightly linked with problems across different cognitive and temperamental domains than is the case in FT children. The absence of group differences in emotional problems is also at odds with previous findings. This could be due to differences in measures used to assess these. Indeed, while a group difference was observed on emotional problems, as measured by the BRIEF-2, with a low to moderate effect size, there was no such difference on with SDQ-rated emeotional problems. A principal component analysis conducted on an overlapping sample of the current participants aged 4 to 7 (Vanes et al., 2021) suggests that, while the BRIEF-2 emotional problem sub-scale score is mostly associated with cognitive abilities, the SDQ adopts a more emotional, affect driven conceptualization of emotional problems. This may explain the discrepant findings, given that children in our VPT sample have significantly poorer cognitive abilities, in turn affecting their socio-emotional and behavioral development (Montagna and Nosarti, 2016).

The observations in the VPT group reflect the presence of a constellation of symptoms commonly reported in VPT populations: poorer socializing abilities, increased internalizing behaviors and poorer attention, which are referred to as the “preterm behavioral phenotype” (Johnson and Marlow, 2011). At first glance, the co-occurrence of executive function and socio-communication difficulties seen in the VPT group may be erroneously interpreted as ASC-like social cognition deficits (Alduncin et al., 2014). However, VPT children may display a unique ASC phenotype, characterized by more predominant difficulties in cognition, attention and socio-emotional processing. Furthermore, despite VPT children exhibiting elevated autistic traits, these do not always reach clinical thresholds (Johnson and Marlow, 2011). Given the isolated and highly clustered SRS-2 subscale scores in the FT network, we tentatively speculate that the SRS-2 in VPT children captures more general behavioral, cognitive, temperamental and socio-emotional difficulties, with a differing etiological pathway to ASC traits or preterm-specific ASC aetiology and symptomatology, rather than more “pure” autistic traits.

The specific network dynamics of individual SRS-2 subscales, the primary variables of interest in our investigation, highlight the way in which ASC-like traits may interact uniquely with other developmental measures in otherwise healthy VPT children. For example, social motivation and social cognition in VPT participants were related to executive functions, in particular working memory, planning and organizing. Notably, the VPT network revealed that the link between working memory and social cognition was not direct, but mediated by verbal comprehension. Social cognition deficits seemed to impact verbal comprehension, which was in turn associated with working memory. Although the direction of effect cannot be ascertained from our analysis, bidirectional mediating effects might be possible. In a large sample of healthy individuals aged 16–91, Froiland and Davison (2020) found that when controlling for other cognitive measures, working memory had no effect on social perception, or the ability to make inferences and form impressions from social interactions. In their study, verbal comprehension had the largest effect on social perception out of all cognitive measures, with increased verbal comprehension difficulties associated with worse social perception. This finding is in line with ASC research in FT adolescents, which highlights poorer verbal comprehension in ASC participants compared to controls, and associated difficulties in social perception (Holdnack et al., 2011). With respect to planning and organizing, Vogan et al. (2018) showed that an improvement in planning and organizing, self-monitoring and initiating behaviors was associated with reduced social deficits in children with autism. However, improvements in emotional control, shift and inhibition were not associated with better social development. These findings are also aligned with our VPT network results, suggesting that VPT children display interactions between ASC traits and other developmental measures that are similar to those observed in clinical ASC groups.

The findings of co-occurring executive function difficulties underlying ASC phenotype and symptomatology in VPT participants is also in line with ASC research in FT, cognitively able children. Pellicano (2010) showed that early executive function skills predicted future abilities in Theory of Mind tasks in children aged four to seven, even when controlling for age and (non)verbal comprehension, thus highlighting the link between socio-cognitive skills and domain-general processes. These findings could be also interpreted in the context of literature revealing associations between cognitive impairments and socio-emotional difficulties in VPT children (Mansson et al., 2014; Rogers et al., 2016). Neuroimaging studies could offer a possible explanation for the overlap between VPT-specific executive function and broader cognitive difficulties and ASC traits, highlighting associations between preterm-related neonatal brain alterations and a later ASC diagnosis (Ure et al., 2016; Eklöf et al., 2019).

Taken together, our findings highlight the underlying role of executive function and temperamental difficulties in the VPT presentation of ASC traits. In particular, our findings suggest that ASC traits, as measured by the SRS-2, may reflect more general developmental difficulties specifically in VPT children, rather than a segregated or “pure” presentation of autistic traits, highlighting the importance of understanding the way in which these traits interact with other developmental measures. This is consistent with recent efforts to develop a cognitive understanding of ASC (Frith, 2021; Happé and Frith, 2021), as a selective focus on behavioral phenotypes could lead to a too broad a definition of ASC, capturing behaviors that are not intrinsically inherent to ASC. Studies focusing on a cognitive understanding of ASC allow for core differences and specificities to be revealed, which in turn reduces the risk of misattributing VPT phenotype-related behaviors to ASC.

Our findings demonstrate the theoretical and practical relevance of departing from diagnostic-driven labels, and focusing on dimensional and transdiagnostic traits, such as executive function deficits, in order to increase our current understanding of psychopathology (Krakowski et al., 2020; Siugzdaite et al., 2020; Vaidya et al., 2020). Our findings further support the concept of equifinality (Cicchetti and Rogosch, 1996), recognizing the possible existence of multiple pathways associated with specific outcomes. We have in fact recently shown that only a subgroup of VPT children exhibiting high ASC traits displayed neonatal cerebellar alterations, suggesting distinct aetiological trajectories associated with ASC outcomes (Hadaya et al., 2022). This study, and in particular our results highlighting the centrality of executive function and behavioral problems in driving other difficulties in VPT children, further strengthens the argument in favor of person-based treatment approaches targeting underlying behaviors and difficulties rather than surface level symptoms (Kasari et al., 2014). This is particularly important when recognizing the potential for these underlying mechanisms, such as poor executive control, to predict later ASC-related difficulties (Pellicano, 2010) and the importance of targeting these mechanisms early in development.

Our study has several limitations. First, since we only considered VPT children, our findings are not generalizable to moderate-to-late preterm children (32–37 weeks’ gestation). Second, the higher prevalence of VPT children reaching a clinical threshold cut-off on the SRS-2, which is parent-rated, compared to other studies, could introduce a rater bias when compared to studies using clinical observations (Aldridge et al., 2012; Treyvaud et al., 2013). This limitation extends beyond the answers on the SRS-2, as all our measures used parental reports for evaluating child behavior, except IQ, which may lead to common method variance bias (Podsakoff et al., 2003). In addition, all measures included in this study were collected at a single time point, making inferences about directionality challenging. Future research can usefully investigate longitudinal network changes over time, in order to fully understand the potential causal dynamics between the behavioral measures under investigation. Finally, the unequal sample sizes of the VPT and FT groups may have contributed to qualitative and quantitative differences in network associations between groups, given that smaller sample sizes are more likely to result in empty networks with non-significant edges (Epskamp, 2016). However, even our smaller (FT) sample was sufficiently powered to establish associations in a comparable range to the VPT group, suggesting that both groups were similarly sensitive to detect associations between nodes. In addition, an explicit comparison of networks was enabled using a bootstrapping procedure, which mitigates the impact of unequal sample sizes.

While this study shows that VPT children are likely at greater risk of displaying ASC traits compared to their FT counterparts, it also highlights that simple group comparisons and focusing on symptom thresholds in isolation, without considering other developmental markers, are not sufficient to fully understand the complexity of the interplay between VPT birth and ASC traits. Our network analysis highlighted underlying constructs leading to ASC behaviors, which may have practical implications, offering a perspective for person-based interventions for VPT children. Indeed, rather than targeting perceived symptoms belonging to specific diagnostic categories, it might be beneficial to focus on their underlying root traits. Future studies can build on these findings by investigating the influence of protective factors that may attenuate the sequelae of VPT birth. We and others have previously shown that supporting parenting and a stimulating home environment promoted resilience against behavioral difficulties in VPT samples (Feldman, 2007; Treyvaud et al., 2009; Ranger et al., 2014; Vinall and Grunau, 2014; Vanes et al., 2021), and may offer useful avenues of further exploration.

## Data availability statement

The raw data supporting the conclusions of this article will be made available by the authors, without undue reservation.

## Ethics statement

The studies involving human participants were reviewed and approved by London South East Research Ethics Committee (REC: 19/LO/1940) and London Stanmore Ethics Committee (REC: 18/LO/0048). Written informed consent to participate in this study was provided by the participants’ legal guardian/next of kin.

## Author contributions

ADE, CN, ML, and LDV: conceptualization. LH, DK, ML, and LDV: methodology. ML and LDV: formal analysis and investigation. ML, LH, LDV, and CN: writing—original draft preparation. ML, LDV, LH, PD, ES, SJC, FH, ADE, and CN: writing—review and editing. CN: supervision. ADE, SJC, PD, ES, and CN: funding acquisition. All authors contributed to the article and approved the submitted version.

## Funding

This work was financially supported by the Medical Research Council (UK) (grant number: MR/S026460/1). The perinatal data analyzed in this study were acquired during independent research funded by the National Institute for Health and Research (NIHR) Program Grants for Applied Research Program (RP-PG-0707-10154).

## Conflict of interest

The authors declare that the research was conducted in the absence of any commercial or financial relationships that could be construed as a potential conflict of interest.

## Publisher’s note

All claims expressed in this article are solely those of the authors and do not necessarily represent those of their affiliated organizations, or those of the publisher, the editors and the reviewers. Any product that may be evaluated in this article, or claim that may be made by its manufacturer, is not guaranteed or endorsed by the publisher.

## Supplementary material

The Supplementary material for this article can be found online at: https://www.frontiersin.org/articles/10.3389/fpsyg.2023.1119196/full#supplementary-material
