# Supplementary material for: Exploring cognitive, behavioral and autistic trait network topology in very preterm and term-born children
Source: Front Psychol. 2023 Apr 27;14:1119196. doi: 10.3389/fpsyg.2023.1119196 (PMC10176608; doi:10.3389/fpsyg.2023.1119196)
Supplement: Supplementary file 1 [file Image_1.pdf]

Supplementary Material

Figure S1:
Correlation matrix for all outcome variables included in the VPT network:

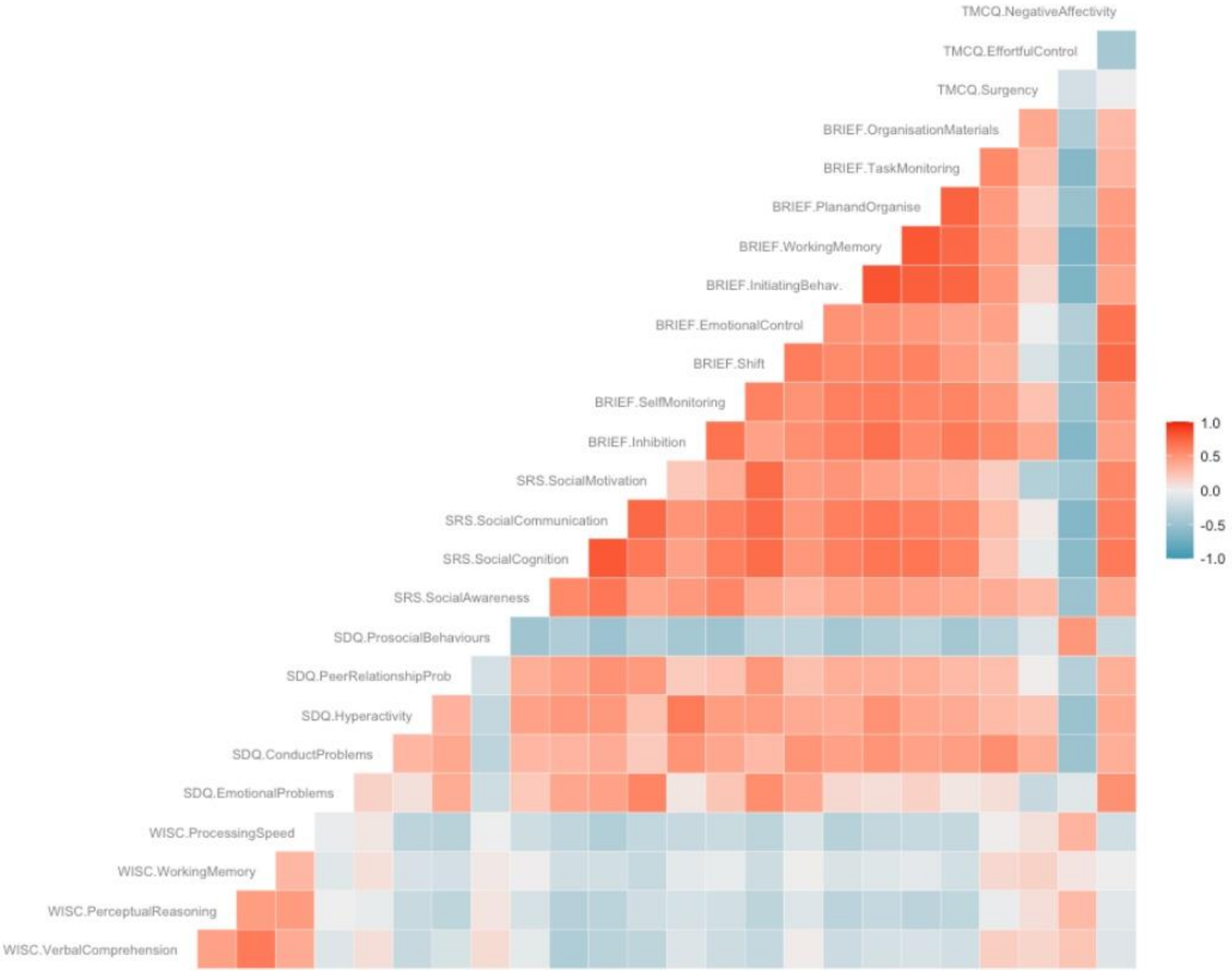

***Note**. WISC: Wechsler Intelligence Scale for Children; SDQ: Strengths and Difficulties questionnaire; SRS-2: Social Responsiveness Scale; BRIEF-2: Behaviour Rating Inventory of Executive Functioning, Second Edition; TMCQ: Temperament in Middle Children Questionnaire*
